# Duodenal fistula secondary to erosion by an expanded polytetrafluoroethylene graft in a living donor liver transplant: a case report and review of the literature

**DOI:** 10.3389/frtra.2026.1799453

**Published:** 2026-06-17

**Authors:** Kausar Makki, Nalini Kanta Ghosh, Yogesh Yadav, Tathagata Karan, Anil Agarwal, Piyush Srivastava, Vivek Vij

**Affiliations:** 1 Department of Liver Transplant and HPB Surgery, Fortis Hospital, Noida, Uttar Pradesh, India; 2 Department of Liver Transplant Anaesthesia and Critical Care, Fortis Hospital, Noida, Uttar Pradesh, India

**Keywords:** duodenal fistula, endoscopy, liver transplant, outcomes, polytetrafluoroethylene graft

## Abstract

Living donor liver transplantation (LDLT) has become an established treatment for patients with decompensated chronic liver diseases of various etiologies, acute liver failure, and liver tumors (primary and secondary). A right lobe graft without the middle hepatic vein is often preferred to achieve adequate graft weight, and an expanded polytetrafluoroethylene (ePTFE) graft is used to reconstruct the anterior sector outflow and optimize allograft function. Generally, this approach is safe and effective until optimal regeneration of the posterior sector occurs. Thrombosis of the ePTFE graft is usually inconsequential for the recipient. Other rare complications of the ePTFE graft include migration into the common bile duct, stomach, duodenum, or jejunum. We report a case of ePTFE graft migration into the duodenum, resulting in a duodenal fistula, which was resolved after endoscopic removal and clipping, in a 44-year-old patient with chronic liver disease secondary to hepatitis B and hepatitis D co-infection. The uniqueness of this case lies in the rarity of this complication and its successful endoscopic management. At 6 months of follow-up, the patient is doing well, with normal allograft function and complete healing of the duodenal fistula. This case highlights the potential complication of an inert ePTFE graft in LDLT, which requires clinical suspicion, evaluation, and can be managed endoscopically.

## Introduction

Living donor liver transplantation (LDLT) is the preferred alternative to deceased donor liver transplantation in regions where deceased donor organs are scarce. Among the various graft options (left lateral graft, left lobe graft with the middle hepatic vein (MHV), and right lobe graft with or without the middle hepatic vein), the right lobe graft without the middle hepatic vein is most commonly procured because it provides optimal graft weight for the recipient while keeping donor safety into consideration. However, these grafts require back-table reconstruction of the anterior sector veins (segment V and segment VIII veins).

Reconstruction of the anterior sector of a right lobe liver graft is important because it increases the effective functional graft volume. This reconstruction is performed on the back table using either portal vein grafts harvested from the explanted liver or cryopreserved vein grafts. In centres where LDLT predominates, the availability of cryopreserved grafts is limited, and many times, in cases like hepatocellular carcinoma or portal vein thrombosis, it may not be prudent to harvest suitable grafts from the explanted liver. Moreover, the retrieval of grafts from the explanted liver is a time-consuming process and may prolong the anhepatic phase. Therefore, many centres rely on synthetic grafts, such as expanded polytetrafluoroethylene (ePTFE, Gore-Tex, W.L. Gore & Associates, Inc., United States). ePTFE grafts are inert, and many studies have demonstrated their safety in liver transplantation (1, 2). Although complications associated with artificial grafts are rare, several adverse events have been reported in the literature, including graft thrombosis, infection, graft stenosis due to neointimal hyperplasia, pseudoaneurysm formation, chronic perigraft seroma, serous leakage, and, rarely, graft migration to surrounding organs. Studies reporting hollow viscus perforation and migration of ePTFE grafts are rare (3). We encountered a similar complication in one of our patients who underwent LDLT using a modified right lobe graft, in which an ePTFE graft had been used to drain anterior sector veins. The patient subsequently developed an enterocutaneous fistula originating from the duodenum. The diagnosis was initially established by imaging (CT scan) and was subsequently confirmed and managed endoscopically.

Relevant case reports and case series have been reviewed (PubMed, Google Scholar) to elucidate the pathogenesis, clinical features, and outcome of such patients. Consent for publication of this case report was obtained from the patient.

## Case report

A 44-year-old man with decompensated chronic liver disease, secondary to hepatitis B and hepatitis D co-infection [Child–Turcotte–Pugh score 11, Model for End-Stage Liver Disease (MELD) score 27], complicated by hepatocellular carcinoma, underwent LDLT. His 20-year-old son was identified as a suitable donor and was considered for donation after evaluation by the hospital committee. He received a right lobe graft without the MHV, and a 6-mm ePTFE graft was used to reconstruct the segment V and VIII veins to form a neo-MHV, which was then anastomosed to the inferior vena cava (IVC) as a conjoint RHV–neo-MHV anastomosis. Post-operatively, doppler ultrasonography of the liver allograft revealed a triphasic outflow. On post-operative day 15, the patient developed fever, and a contrast-enhanced CT scan (CECT) of the abdomen demonstrated a fluid collection near the cut surface of the allograft (Figure 1A). A sonographic-guided percutaneous drainage (PCD) of the purulent fluid was performed. Following drainage, the patient showed clinical improvement and was discharged in a stable condition on day 21. The drain output gradually decreased, and the PCD was removed. He remained asymptomatic for 6–8 months before developing an erythematous swelling and sinus in the epigastric region that drained purulent fluid (Figure 2A). His biochemical parameters and imaging studies ruled out any intra-abdominal collection or biliary complication. Two years after liver transplantation, he noticed the passage of ingested food particles through the sinus tract, indicating an enterocutaneous fistula originating from the stomach or duodenum. Considering the time lag between the insertion of the PCD for the drainage of the post-transplant collection and the development of the fistula, a PCD-induced iatrogenic duodenal injury was considered less likely. He underwent upper gastrointestinal endoscopy, which showed duodenal perforation by the ePTFE graft. The graft was removed endoscopically, and the defect was approximated using an endoclip (Figures 3A–C). The patient had no peritonitis or other complications. His fistula output decreased significantly after removal of the ePTFE graft, and the fistula started healing spontaneously (Figure 2B). Follow-up MRI showed a normal right hepatic vein with no adjacent collections (Figure 1B). At 6 months following endoscopic removal of the ePTFE graft, the patient remains healthy, with liver function tests within the normal range and an almost completely healed fistula tract that is no longer draining. Figure 4 shows a timeline of events in this patient.

**Figure 1:**
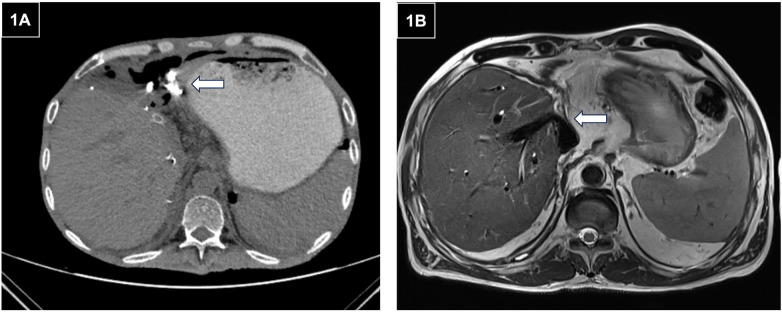
Radiological image **(1A)**: CECT during immediate post-transplant collection with PCD insitu and it also shows normal position of the ePTFE graft, **(1B)**: Post- ePTFE graft removal follow-up MRI showing normal right hepatic vein, no residual collection.

**Figure 2:**
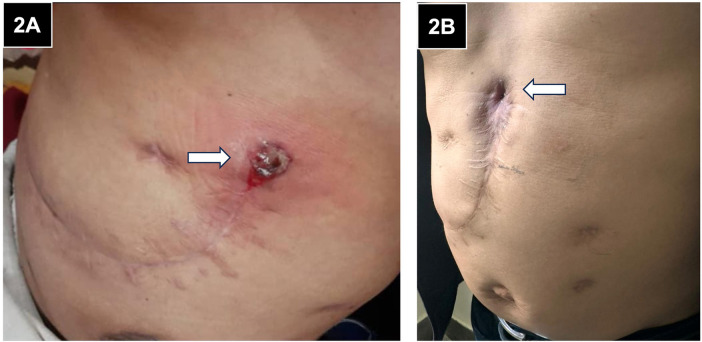
Clinical images **(2A)**: showing red and discharging sinus in the epigastric surgical site, **(2B)**: showing healed fistula site.

**Figure 3:**
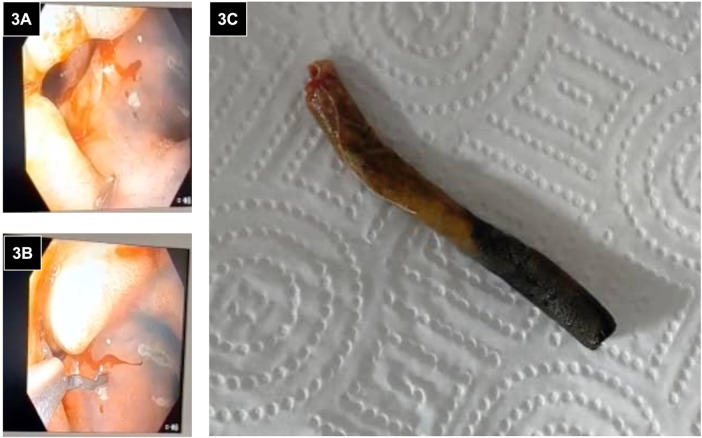
Endoscopic images **(3A)**: showing the duodenal end of the ePTFE graft, **(3B)**: duodenal fistula site approximation using endo clip, **(3C)**: Retrieved ePTFE graft.

**Figure 4:**
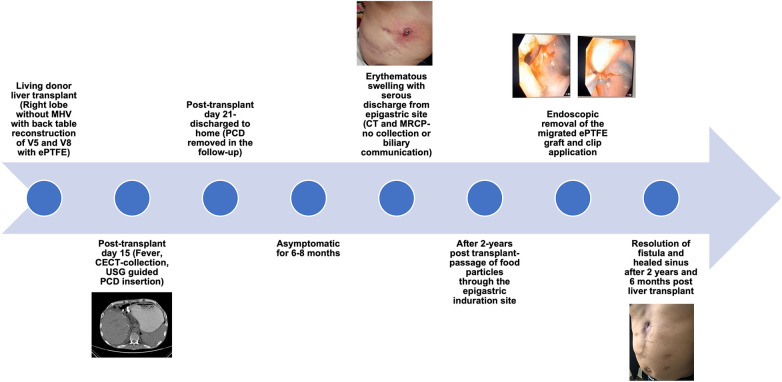
Diagram showing timeline of events occurred in the patient.

## Discussion

During right lobe LDLT, the MHV is not included in the graft to ensure donor safety. Hence, the venous drainage of the anterior sector (segments V and VIII) needs to be reconstructed on the back table to prevent anterior sector congestion and to provide better functional volume to the recipient, especially in cases of marginal grafts or a low graft weight-to-recipient weight ratio (GRWR) (1, 4). The concept of an adequate GRWR varies among patients and is determined largely by the quality of the donor liver, the recipient’s MELD score, and intra-operative and post-operative outcomes. Various materials are used to reconstruct anterior sectoral veins. However, being LDLT-predominant, our centre, like most other eastern centres, relies on the use of ePTFE grafts. This not only ensures an ‘always available’ graft but also keeps the anhepatic time to a minimum (5). We have used ePTFE grafts for anterior sector reconstruction in over 2,500 cases and also for cavoportal hemitransposition and IVC reconstruction in patients with Budd–Chiari syndrome, with no PTFE-related complications. Our technique for the use of ePTFE grafts has been described elsewhere (6). A few studies have reported rare complications, such as fistula formation, ePTFE graft migration, and gastric or duodenal perforation. Ringed ePTFE grafts have been associated with a higher incidence of complications such as duodenal perforation, likely due to the presence of rings around the graft (7). In our case, however, we used a non-ringed ePTFE graft.

These patients may present with clinical evidence of infection (pain, fever), jaundice [due to common bile duct (CBD) involvement] (8), or dyspepsia (stomach or duodenal involvement). A few patients may remain asymptomatic, with the complication incidentally detected on follow-up imaging (9–11). In our case, the patient presented with a chronic sinus that evolved into a duodenocutaneous fistula, which has not been reported previously.

Studies evaluating ePTFE complications have found several risk factors; early thrombosis of the ePTFE graft may cause local inflammation and subsequent adhesion formation (3). Patients who have undergone multiple surgeries with abdominal adhesions may be at an increased risk of ePTFE adhesion to the surrounding viscus, facilitating graft migration (8). Also, post-operative bile leaks or other perihepatic collections, such as hematomas, may not only cause ePTFE thrombosis but also distort the shape of the graft. The graft may become embedded within the adhesions that develop. Interventional procedures, such as placement of a percutaneous drain, may further aggravate this process and lead to graft injury or displacement if not performed carefully (11, 12). In our patient, we had to place a percutaneous drain during the immediate perioperative period, which may have been an inciting factor for duodenal adhesion formation and subsequent ePTFE erosion into the duodenum.

The diagnosis is often established on imaging, which can demonstrate a thrombosed graft, graft displacement or migration, air foci around the graft, and thickening of the adjacent bowel wall (12). In some cases, the diagnosis is made during endoscopic evaluation, where the graft may be seen protruding partially or completely, observed as in this patient (8).

A review of the literature revealed only a few case reports and three case series describing PTFE graft-induced perforation of a hollow viscus (bile duct or gastrointestinal tract) (Table 1). Patients may present with signs and symptoms of sepsis, requiring urgent invasive treatment (surgical, endoscopic, or percutaneous). However, in some cases where patients were stable, conservative management with interval assessment has been performed to ensure passage of the graft (Table 1). Patients in whom the graft had invaded the common bile duct were managed with endoscopic or percutaneous dilatation of the biliary stricture (8), and in one patient, the graft was removed percutaneously (12). Endoscopic removal is possible when the graft is accessible and there is no free peritoneal communication. One patient in the series by Woo et al. was managed with endoscopic graft removal (8). Similarly, in our case, the graft was removed endoscopically, and the defect was approximated using an endoclip, resulting in closure of the fistula.

**Table 1:** Published cases on hollow viscus perforation by the ePTFE graft in liver transplantation.

Author (year)	Number of cases	Type of hollow viscus perforation	Duration between surgery and migration (months/range)	Management	Outcome
Kim et al. (2011) (12)	4	Stomach/duodenum: 3 CBD: 1	23–34	No treatment: 3 Percutaneous removal: 1	3 survived 1 dead
Ha et al. (2014) (9)	1	Stomach	6	Surgical removal: 1	Alive
Hung (2015) (14)	1	Duodenum	6	Surgical removal	Alive
Hsu et al. (2017) (3)	3	Duodenum	5,12,13	Surgical removal: 3	2 alive 1 dead
Esposito et al. (2017) (15)	1	Stomach	132	Surgical removal	Alive
Sultan et al. (2018) (11)	1	Duodenum	30	No treatment (spontaneous expulsion)	Alive
Woo et al. (2021) (8)	14	CBD: 3 Stomach: 3 Duodenum: 5 Jejunum: 5	8–128	Surgical removal: 1 Endoscopic removal: 1 No treatment: 9 ERC: 3	13 alive 1 needed retransplantation
Saraswathy et al. (2024) (10)	1	Stomach	48	No treatment (spontaneous expulsion)	Alive
Egeli et al. (2025) (13)	1	Duodenum	96	No treatment (spontaneous expulsion)	Alive
Our case	1	Duodenum	28	Endoscopic removal	Alive

CBD, common bile duct; ERC, endoscopic retrograde cholangiography.

Cases of hollow viscus perforation due to ePTFE grafts may be associated with significant morbidity and can require emergency exploratory laparotomy (3). One case report has also reported spontaneous expulsion of the ePTFE graft from the body over time without any adverse events for the recipient (10, 13). In our patient, a cutaneous fistula tract developed from the site of duodenal erosion by the ePTFE. Following endoscopic removal of the migrated ePTFE, the fistula began to heal on its own. The patient experienced no further symptoms and continues to do well on follow-up.

To prevent such complications, some centres recommend placing an omental patch over the ePTFE graft to act as a buffer (3). Also, when placing percutaneous drains in the post-operative period, it is important to be aware of the location of the ePTFE graft and to avoid injury to it.

## Conclusion

This case report highlights the rare complication of ePTFE graft migration into a hollow viscus (the duodenum in this case). Transplant surgeons using these grafts should be aware of and vigilant for this potential complication. When expertise is available, it can be managed endoscopically.

## Data Availability

The original contributions presented in the study are included in the article/Supplementary Material; further inquiries can be directed to the corresponding authors.
